# Left atrial longitudinal strain by speckle tracking echocardiography correlates well with left ventricular filling pressures in patients with heart failure

**DOI:** 10.1186/1476-7120-8-14

**Published:** 2010-04-21

**Authors:** Matteo Cameli, Matteo Lisi, Sergio Mondillo, Margherita Padeletti, Piercarlo Ballo, Charilaos Tsioulpas, Sonia Bernazzali, Massimo Maccherini

**Affiliations:** 1 Department of Cardiovascular Diseases, University of Siena, Siena, Italy; 2 Cardiology Operative Unit, S. Maria Annunziata Hospital, Firenze, Italy; 3 Department of Cardiothoracic Surgery, University of Siena, Italy

## Abstract

**Background:**

The combination of early transmitral inflow velocity and mitral annular tissue Doppler imaging (E/Em ratio) is widely applied to noninvasively estimate left ventricular (LV) filling pressures. However E/Em ratio has a significant gray zone and its accuracy in patients with heart failure is debated. Left atrial (LA) deformation analysis by speckle tracking echocardiography (STE) was recently proposed as an alternative approach to estimate LV filling pressures. This study aimed at exploring the correlation of LA longitudinal function by STE and Doppler measurements with direct measurements of LV filling pressures in patients with heart failure.

**Methods:**

A total of 36 patients with advanced systolic heart failure (ejection fraction ≤35%), undergoing right heart catheterization, were studied. Simultaneously to pulmonary capillary wedge pressure (PCWP) determination, peak atrial longitudinal strain (PALS) and mean E/Em ratio were measured in all subjects by two independent operators. PALS values were obtained by averaging all segments (global PALS), and by separately averaging segments measured in the 4-chamber and 2-chamber views.

**Results:**

Not significant correlation was found between mean E/Em ratio and PCWP (R = 0.15). A close negative correlation between global PALS and the PCWP was found (R = -0.81, p < 0.0001). Furthermore, global PALS demonstrated the highest diagnostic accuracy (AUC of 0.93) and excellent sensitivity and specificity of 100% and 93%, respectively, to predict elevated filling pressure using a cutoff value less than 15.1%. Bland-Altman analysis confirmed this close agreement between PCWP estimated by global PALS and invasive PCWP (mean bias 0.1 ± 8.0 mmHg).

**Conclusion:**

In a group of patients with advanced systolic heart failure, E/Em ratio correlated poorly with invasively obtained LV filling pressures. However, LA longitudinal deformation analysis by STE correlated well with PCWP, providing a better estimation of LV filling pressures in this particular clinical setting.

## Background

Accurate noninvasive estimation of left ventricular (LV) filling pressures is a clinical valuable tool to predict the severity of different heart diseases and to decide therapeutic strategy, particularly in patients with heart failure [1]. In fact, invasive capillary wedge pressure (PCWP) measurement, a surrogate for LV filling pressures, is directly associated with functional capacity and prognosis in patients with heart failure [2-4]. Several echocardiographic indices have been proposed to assess LV filling pressures. In particular, early transmitral flow velocity (E) combined with mitral annular early diastolic velocity (Em) derived from tissue Doppler imaging (E/Em ratio) has been shown to correlate with PCWP in a wide range of cardiac patients [5-9]. However E/Em ratio has a significant gray zone [5,7,10] and its accuracy, particularly when applied in patients with heart failure, is debated [11,12]. Speckle tracking echocardiography (STE) is a novel non-Doppler-based method for the angle-independent and objective quantification of myocardial deformation from standard bidimensional datasets [13-16]; in contrast to Doppler-derived indexes, speckle tracking has the advantage of being angle-independent, and to be less affected by reverberations, side lobes and drop out artifacts. STE has recently evolved and, enabling the quantification of longitudinal myocardial left atrial (LA) deformation dynamics, it was recently proposed as an alternative approach for the LV filling pressure estimation[17]. Therefore this study aimed at exploring the utility of these Doppler and LA STE derived echocardiographic indices in predicting LV filling pressures in consecutive patients with systolic heart failure undergoing right heart catheterization.

## Methods

### Study population

Forty-eight consecutive patients with symptomatic chronic (> 6 months) systolic heart failure (ejection fraction ≤ 35% and New York Heart Association class III to IV symptoms), who underwent a right heart catheterization, in the cardiac catheterization laboratory (n = 34) or in the intensive care unit (n = 14), because of concerns about hemodynamic derangements and/or to a staging of patients listed for heart transplantation, were enrolled. All were in sinus rhythm, hemodinamically stable and had simultaneous right heart catheterization and transthoracic echocardiographic imaging. A previous cardiac resynchronization therapy with defibrillator (CRT-D) was not an exclusion criteria. Patients were excluded if they had nonsinus rhythm, mechanical ventilation, severe mitral regurgitation, any mitral stenosis, any prosthetic mitral and/or aortic valve, heart transplantation or an insufficient imaging quality of the LA endocardial border. All subjects gave their written informed consent for the participation to the study. All work was in compliance with the declaration of Helsinki and it was performed with the approval of local ethics committee.

### Cardiac catheterization

Readings of invasive cardiac pressure measurements were performed by an investigator blinded to all echocardiographic data. The pressure transducers were balanced before data acquisition with the zero level at mid-axillary line. Pulmonary artery (PA) catheters were used to measure PA pressures, mean right atrial pressure and mean PCWP. The wedge position was verified by fluoroscopy, phasic changes in pressure waveforms and oxygen saturation. Cardiac output and cardiac index were derived by the thermodilution technique (average of 5 cardiac cycles with < 10% variation) and by the Fick equation through sampling of a mixed central venous blood gas taken in the pulmonary artery and of an arterial blood gas.

### Standard echocardiography

Echocardiographic studies were performed using a portable echocardiograph (Vivid i, GE, USA), equipped with a 2.5 MHz transducer. Subjects were studied in a supine position during the heart catheterization. Measurements of left ventricular and left atrial dimensions, LV ejection fraction, and diastolic LV filling velocities were made in accordance with current recommendations of ASE [18]. LV ejection fraction, measured using Simpson's method, was used as a standard index of global LV systolic function. The ratio between peak early (E) and late (A) diastolic LV filling velocities was used as standard indices of LV diastolic function [19]. LA volumes were measured using the area-length method, from the apical four and two chamber views. LA volumes were subsequently indexed by body surface area (BSA). The time interval between the onset of the QRS on the electrocardiogram and the aortic and mitral valve opening and closure were measured using pulsed-wave Doppler from the LV outflow and inflow, respectively.

### Tissue Doppler Imaging

LV longitudinal function was explored by pulsed Tissue Doppler imaging, placing the sample volume at the level of mitral lateral and septal annulus from the apical four-chamber view [20]. Mean peak systolic (Sm), early diastolic (Em), and late diastolic (Am) annular velocities were obtained by averaging respective values measured at the septal and lateral sides of the mitral annulus. Mean Em and the derived mean Em/Am ratio were used as load-independent markers of ventricular diastolic relaxation [21]. Mean E/Em ratio was also calculated [7].

### Speckle Tracking Echocardiography

For speckle tracking analysis, apical four- and two-chamber views images were obtained using conventional two dimensional gray scale echocardiography, during breath hold and with a stable ECG recording. Care was taken to obtain true apical images using standard anatomic landmarks in each view and not foreshorten the left atrium, allowing a more reliable delineation of the atrial endocardial border. We also avoided visualization of the LA appendage in the apical 2-chamber view to minimize its effect on LA strain measurements. Three consecutive heart cycles were recorded and averaged. The frame rate was set between 60 and 80 frames per second.

**Figure 1:**
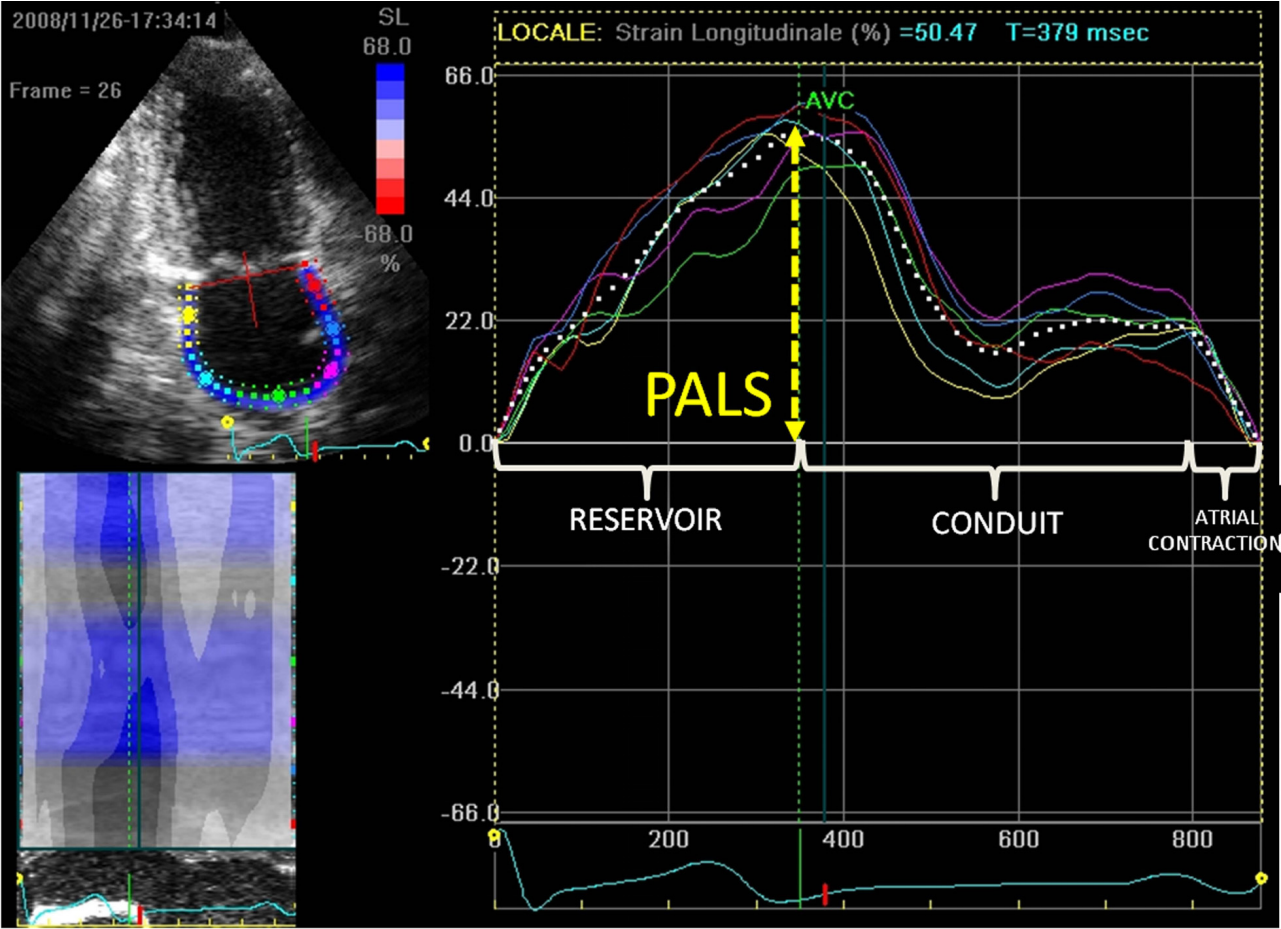
**Measurement of peak atrial longitudinal strain (PALS) from an apical two-chamber view**. The dashed curve represents the average atrial longitudinal strain along the cardiac cycle. (PALS, peak atrial longitudinal strain; AVC, aortic valve closure).

The analysis of files recorded was performed off-line by a single experienced and independent echocardiographer, who was not directly involved in the image acquisition and had no knowledge of hemodynamic mesasurements, using a commercially available semi-automated two-dimensional strain software (EchoPac, GE, USA). As previously described [13], LA endocardial border is manually traced in both four- and two-chamber views, thus delineating a region of interest (ROI), composed by 6 segments. Then, after the segmental tracking quality analysis and the eventual manual adjustment of the ROI, the longitudinal strain curves are generated by the software for each atrial segment. As shown in Figure 1, peak atrial longitudinal strain (PALS), measured at the end of the reservoir phase, was calculated by averaging values observed in all LA segments (global PALS), and by separately averaging values observed in 4- and 2-chamber views (4- and 2-chamber average PALS, respectively). The time to peak longitudinal strain (TPLS) was also measured as the average of all 12 segments (global TPLS) and by separately averaging values observed in the two apical views (4- and 2-chamber average TPLS). In patients in whom some segments were excluded because of the impossibility of achieving adequate tracking, PALS and TPLS were calculated by averaging values measured in the remaining segments.

### Reproducibility

To assess the reproducibility of global PALS, 10 patients were randomly selected: Bland-Altman analysis was performed to evaluate the intra- and interobserver agreement by repeating the analysis 1 week later by the same observer and a second independent observer. Bland-Altman analysis demonstrated good intra- and interobserver agreement, with small bias not significantly different from zero. Mean differences ± 2 standard deviations were 0.4 ± 2.1% and 0.6 ± 3.4%, for intra- and interobserver agreement, respectively.

### Statistical analysis

Data are shown as mean ± SD. A P value < 0.05 was considered statistically significant. Pearson's correlation coefficients were calculated to assess the relationships between continuous variables. On the basis of similar previous studies [22-25], a PCWP value of 18 mmHg was chosen as the cutoff value. Sensitivity and specificity were calculated using standard definitions, receiver operating characteristic curves were constructed and the area under the curve was calculated for the prediction of PCWP ≥18 mmHg. The agreement between different methods was assessed with the method of Bland and Altman [26]. Analyses were performed using the SPSS (Statistical Package for the Social Sciences, Chicago, Illinois) software Release 12.0.

## Results

### Patients characteristics

Of 48 patients screened, 36 patients (15 women, 21 men) met eligibility criteria during the study period. The admitting diagnosis were coronary artery disease (29 patients) and non-ischemic cardiomiopathy (7 patients). All patients were classified as New York Heart Association class III to IV with a mean LV ejection fraction of 26.1 ± 5%. Five were excluded for nonsinus rythm; 3, for severe mitral valve disease, 3 for poor echocardiographic window; and 2, for difficulties in heart catheterization. Table 1 shows the clinical, echocardiographic and catheterization data between the 15 patients with PCWP<18 mmHg and the 21 patients with PCWP ≥18 mmHg. As listed in Table 1, there were no significant differences in such characteristics as age, hypertension, diabetes, blood pressure, heart rate, presence of coronary artery disease and medical therapy between patients with PCWP<18 mmHg and ≥18 mmHg. Patients with previous CRT-D implant were all in spontaneous sinus rhythm and were equally distributed in the two groups.

**Table 1:** Clinical, echocardiographic and catheterization data of the study population (n = 36)

	PCWP <18 mmHg (n = 15)	PCWP ≥18 mmHg (n = 21)	p Value
***Clinical data***			
**Age**	57.7 ± 8.1	57.3 ± 8.6	0.77
**Gender **(% female)	43.2	42.3	0.65
**Body surface area **(m^2^)	1.95 ± 0.2	1.94 ± 0.2	0.70
**Hypertension**	20 (89%)	12 (88%)	0.66
**Diabetes mellitus**	8 (36%)	5 (35%)	0.81
**Hypercholesterolemia**	17 (77%)	10 (75%)	0.51
**Current smoker**	2 (9%)	2 (12%)	0.44
**Ischemic cause **(%)	81%	82%	0.65
**CRT-D**	4 (28.6%)	7 (33.3%)	0.21
***Medical therapy***			
**Ace-inhibitors or ARB**	10 (66.6%)	14 (66.6%)	0.81
**Beta-blockers**	8 (53.3%)	11 (52.3%)	0.66
**Spironolactone**	7 (46.7%)	9 (42.8%)	0.22
**Loop diuretics**	13 (86.7%)	18 (85.7)	0.44
**Statins**	9 (60.0%)	14 (66.7%)	0.32
***Echocardiographic data***			
**Left atrial area **(cm^2^)	26.9 ± 5.9	33.1 ± 6.6	0.02
**Left atrial volume indexed **(ml/m^2^)	30.2 ± 9	41.1 ± 10.1	0.009
**End-diastolic LV diameter **(mm)	57.3 ± 8.3	63.0 ± 8.0	0.01
**LV mass index **(g/m^2^)	116.6 ± 31.3	118.3 ± 33.5	0.21
**LV Ejection Fraction **(%)	26.5 ± 3.5	25.7 ± 4.3	0.15
**Mitral E **(cm/s)	82.6 ± 33	99.4 ± 46	<0.001
**Mitral E/A ratio**	2.19 ± 1.1	3.66 ± 1.3	<0.001
**Sm **(cm/s)	5.0 ± 1.1	5.0 ± 1.2	0.81
**Em **(cm/s)	6.6 ± 1.8	6.2 ± 1.8	0.24
**E/Em **(cm/s)	12.6 ± 6.4	15.9 ± 7.9	0.01
**Global PALS **(%)	16.9 ± 4.0	9.8 ± 4.2	<0.001
**4-chamber PALS **(%)	14.3 ± 3.8	8.0 ± 3.6	<0.001
**2-chamber PALS **(%)	18.0 ± 4.6	11.2 ± 4.5	<0.001
**Global TPLS **(ms)	445 ± 81	410 ± 78	0.09
***Catheterization data***			
**Heart rate **(bpm)	73.3 ± 15.3	76.0 ± 15.1	0.20
**Systolic blood pressure **(mmHg)	118 ± 19	116 ± 21	0.28
**Diastolic blood pressure **(mmHg)	79 ± 11	76 ± 13	0.27
**Mean PAP **(mmHg)	21.8 ± 7.8	32.1 ± 9.9	<0.001
**PCWP **(mmHg)	13.1 ± 5.1	23.8 ± 7.4	<0.001
**CI therm **(ml/min/m^2^)	2.12 ± 0.7	2.13 ± 0.9	0.40
**CI fick **(ml/min/m^2^)	1.99 ± 0.4	1.97 ± 0.4	0.42

(PCWP, Pulmonary capillary wedge pressure; CRT-D, previous cardiac resynchronization therapy with defibrillation implantion; ACE, angiotensin-converting enzyme; ARB, angiotensin receptor blocker; LV, left ventricular; E, early transmitral flow velocity; A, atrial transmitral flow velocity; Sm, systolic mitral annular velocity; Em, early diastolic mitral annular velocity; PALS, peak atrial longitudinal strain; TPLS, time-to-peak atrial longitudinal strain; PAP, pulmonary arterial pressure; CI therm, cardiac index estimated by thermodilution; CI fick, cardiac index estimated by the Fick equation.)

### Invasive hemodynamic and echocardiographic measurements

Conventional Doppler and tissue-Doppler indices showed increased mitral E (99.4 ± 46 cm/s vs 82.6 ± 33 cm/s, p < 0.001), mitral E/A ratio (3.66 ± 1.3 vs 2.19 ± 1.1, p < 0.001) and mean E/Em ratio (15.9 ± 7.9 vs 12.6 ± 6.4, p = 0.01) in the patients with PCWP ≥18 mmHg, instead no differences between groups were found for mitral Em. Regarding novel atrial STE measurements, among a total of 432 segments analyzed, the software was able to correctly track 411 (95.1%) segments. Global PALS was significantly lower in patients with PCWP ≥18 mmHg (9.8 ± 4.2% vs 16.9 ± 4.0%, p < 0.001). Similar results were obtained in PALS measured in four-chamber view (8.0 ± 3.6% vs 14.3 ± 3.8%, p < 0.001) and in two-chamber view (11.2 ± 4.5% vs 18.0 ± 4.6%, p < 0.001). No significant differences of TPLS values were detected between two groups.

### Correlation between echocardiographic and invasive measurements

**Figure 2:**
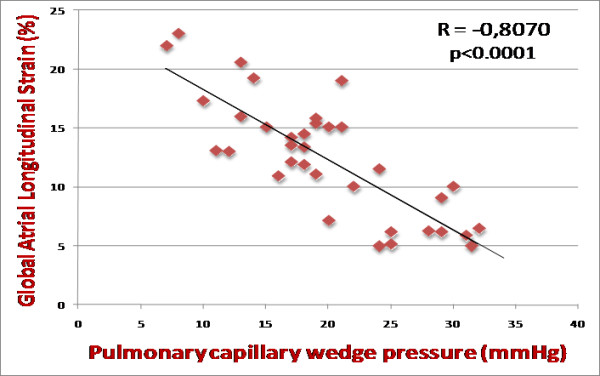
**Correlation between global peak atrial longitudinal strain (PALS) and pulmonary capillary wedge pressure**. R = -0.8070; p < 0.0001. (PALS, peak atrial longitudinal strain).

**Figure 3:**
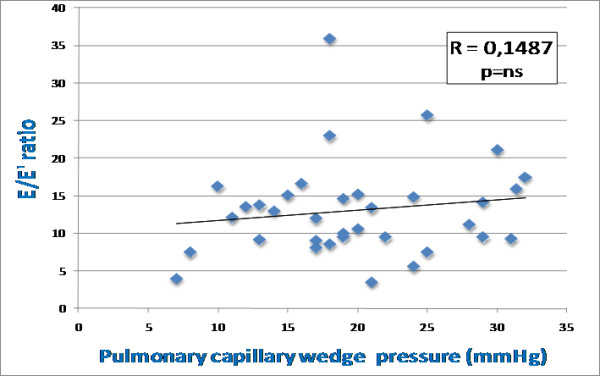
**Correlation between global peak atrial longitudinal strain (PALS) and mean E/Em ratio**. R = 0.1487; p = ns. (PALS, peak atrial longitudinal strain; E, early transmitral flow velocity; Em, early diastolic mitral annular velocity).

A strong inverse correlation was observed between PCWP and global PALS (r = -0.81; p < 0.0001) (Figure 2), instead no correlation was found with mean E/Em ratio (r = 0.15) (Figure 3). Several other significant relations were noted between PCWP and LA volume indexed (r = 0.38; p < 0.01), LA area (r = 0.33; p = 0.021), LV end-diastolic volume (r = 0.23; p = 0.05) and Doppler (mitral E: r = 0.40, p < 0.01; mitral E/A ratio: r = 0.52, p < 0.001) measurements. To better understand the limitation and the lack of correlation between mean E/Em ratio and PCWP in the study population, clinical, echocardiographic and hemodynamic variables were compared between two groups according to the presence or absence of concordant mitral E/Ea ratio > 15 and PCWP > 18 mm Hg. Among all variables analyzed, LV ejection fraction, LV end-diastolic volume, LV end-diastolic diameter and cardiac index appeared statistically different between two groups; the group of patients with discordant E/Em and PCWP measurements presented larger LV dimensions and more impaired systolic function.

### Diagnostic accuracy of noninvasive estimate of elevated filling pressure

**Figure 4:**
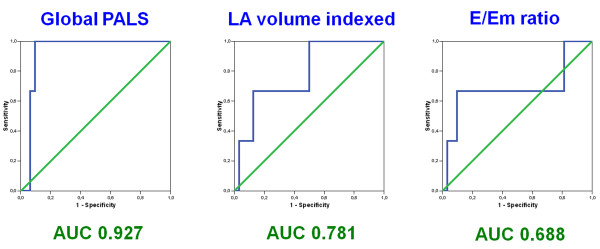
**Diagnostic accuracy of noninvasive estimate of elevated filling pressure**. Receiving operating characteristic (ROC) curves for global peak atrial longitudinal strain (PALS), left atrial (LA) volume indexed and mean E/Em ratio for prediction of pulmonary capillary wedge pressure ≥18 mmHg in patients with advanced systolic heart failure. (PALS, peak atrial longitudinal strain; LA, left atrial; E, early transmitral flow velocity; Em, early diastolic mitral annular velocity; AUC, area under the curve).

**Figure 5:**
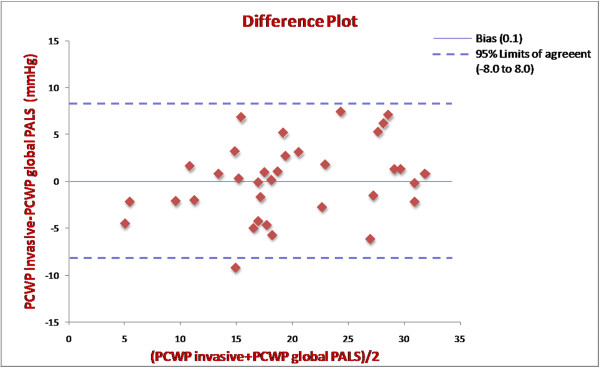
**Bland-Altman analysis**. Bland-Altman agreement plot comparing pulmonary papillary wedge pressure (PCWP) estimated by global peak atrial longitudinal strain (PALS) and invasive PCWP in the 36 patients. Bias (0.1); 95% Limits of agreement (-8.0 to 8.0). (PALS, peak atrial longitudinal strain; PCWP, pulmonary capillary wedge pressure).

To further investigate the value of these echocardiographic indices to predict an elevated filling pressure, we performed receiving operating characteristics (ROC) curve analyses. Area under the curve (AUC), optimal cutoff values and corresponding sensitivities and specificities to predict PCWP 18 mmHg or greater are presented in Table 2. Among all echocardiographic parameters analyzed, global PALS showed the highest diagnostic accuracy (AUC = 0.93) and excellent sensitivity and specificity of 100% and 93.2%, respectively, to predict elevated filling pressure using a cutoff value less than 15.1%. LA volume indexed presented weaker accuracy with an AUC of 0.78 and sensitivity and specificity of 67.9% and 87.5%, respectively, at a cutoff value greater than 37.8 ml/m^2^. Mean E/Em ratio presented limited diagnostic accuracy (AUC = 0.69) (Figure 4). Bland-Altman analysis comparing PCWP estimated by global PALS and invasive PCWP demonstrated a close agreement with a mean bias of 0.1 ± 8.0 mmHg (mean ± 2 standard deviation) (Figure 5).

**Table 2:** Receiver operating characteristics analysis of echocardiographic parameters to predict pulmonary capillary wedge pressure ≥18 mmHg

	Cutoff value	Sensitivity (95% CI)(%)	Specificity (95% CI)(%)	AUC
***Two-dimensional Echo***				
**LV EDV**	>278 ml	50.0 (15.6-85.6)	88.2 (46.3-99.6)	0.60
**LA area**	>36.5 cm^2^	60.0 (32.4-84.1)	50.0 (16.1-85.3)	0.68
**LA volume indexed**	>37.8 ml/m^2^	67.9 (53.9-96.8)	87.5 (56.9-95.7)	0.78
***Mitral flow Doppler***				
**Mitral E wave**	> 94.2 cm/s	90.1 (66.8-99.7)	66.8 (26.3-93.2)	0.72
**E/A ratio**	>2.6	89.4 (65.1-97.2)	65.2 (24.3-92.9)	0.70
***Tissue Doppler***				
**Mean E/Em ratio**	>16.8	67.9 (27.8-91.5)	93.1 (76.9-99.8)	0.69
***Speckle tracking***				
**Global PALS**	<15.1%	100 (83.9-100)	93.2 (78.1-99.8)	0.93

(AUC, Area under the curve; CI, confidence interval; LV, left ventricular; EDV, end diastolic volume; LA, left atrial; E, early transmitral flow velocity; A. atrial transmitral flow velocity; Em, early diastolic mitral annular velocity; PALS, peak atrial longitudinal strain).

## Discussion

In this study we analyzed for the first time the correlation with PCWP of a novel speckle tracking index, the LA longitudinal strain, comparing it with other Doppler indices, in particular the E/Em ratio, in patients with advanced systolic heart failure referred for right heart catheterization. Using simultaneous measured echocardiographic and invasive hemodynamic variables, we found in this particular group of patients a strong correlation between global PALS and PCWP, in contrast with the weaker and not significant correlation showed by mean E/Em ratio. Furthermore, global PALS demonstrated the highest diagnostic accuracy (AUC of 0.93) and excellent sensitivity and specificity of 100% and 93%, respectively, to predict elevated filling pressure using a cutoff value less than 15.1%. ROC curve analysis indicated that, by using this cutoff value of 15.1%, although the relative limited sample size, no one patient in the group of elevated PCWP was misdiagnosed. Bland-Altman analysis confirmed this close agreement between PCWP estimated by global PALS and invasive PCWP. The atrial reservoir phase is essential for LV filling by storing energy during ventricular systole, that is released after MV opening [27]; in our study the global PALS, parameter for the functional evaluation of the atrial reservoir phase (Figure 1), resulted progressively decreased with the augmentation of LV filling pressures. The potential mechanism of this inverse correlation could be explained by the principle that PCWP is the afterload of LA function; if PCWP is high, the left atrium should be chronically stressed, resulting in decrease of LA reservoir function and finally in remodeling with LA chamber dilation, as demonstrated in patients with heart failure [28].

Regarding Doppler indices, mitral E and E/A ratio were significantly higher in the patients with elevated PCWP, but their diagnostic accuracy were relatively poor (AUC of 0.72 and 0.70, respectively). Similarly, mean E/Em ratio appeared unable to adequately predict elevated filling pressure (AUC of 0.69). According to our results, that are confirmed by previous studies [12,29-31], we did not confirm the previously reported finding that changes in the mitral E/Em ratio accurately track changes in PCWP in advanced systolic heart failure [10]. The lack of correlation between mean E/Em ratio and PCWP in our study, particularly evident in patients with larger LV volumes, more impaired cardiac indices, is probably due to the fact that patients with advanced heart failure often have severe LV fibrosis, stiffness and impaired cardiac systolic function, that could restrict systolic and subsequently early diastolic mitral excursion and affect mitral inflow velocity differently than expected, invalidating the E/Em approach in this particular clinical setting.

### Limitations

The measurement of global PALS requires more capability and is contingent on the presence of adequate apical views. However, in this study, which included supine patients on the cardiac catheterization table or in the intensive care unit, the feasibility was excellent at 94%. Considered the close dependence of STE with the single-cardiac cycle strain analysis, it was not possible to conduct the study even in patients with non-sinus rhythm. Regional wall motion abnormalities in severely dilated and/or ischemic ventricles might have altered mitral Em acquisition. However, instead of analyzing only the septal or the lateral Em, we considered the average of both walls [32]. No invasive hemodynamic measurements of LA or LV end-diastolic pressure were performed, considering that PCWP is accepted as a well validated surrogate [33,34].

Although there was a limited sample size, we were able to show novel findings. Nonetheless, larger prospective validation studies using these novel indices are of utmost importance.

## Conclusion

In patients with advanced systolic heart failure mean E/Em ratio may not be a useful index to estimate filling pressures; the measurement of global PALS, instead, provides a close prediction of PCWP and could be considered a promising noninvasive index to assess LV filling pressures in this particular clinical setting.

## Competing interests

The authors declare that they have no competing interests.

## Authors' contributions

MC, ML, MP, CT and SB were responsible for the collection of data; MC drafted the manuscript and performed the statistical analysis; SM and MM were responsible for the design of the study and revised the manuscript for important intellectual content. PC also revised the manuscript for important intellectual content. All authors read and approved the final manuscript.
